# Modulation of Hyaluronan Synthesis by the Interaction between Mesenchymal Stem Cells and Osteoarthritic Chondrocytes

**DOI:** 10.1155/2015/640218

**Published:** 2015-07-26

**Authors:** Eliane Antonioli, Carla A. Piccinato, Helena B. Nader, Moisés Cohen, Anna Carla Goldberg, Mario Ferretti

**Affiliations:** ^1^Hospital Israelita Albert Einstein, No. 627/701, 05652-900 São Paulo, SP, Brazil; ^2^Department of Biochemistry, Molecular Biology Program, Federal University of São Paulo, No. 100, 04044-020 São Paulo, SP, Brazil; ^3^Orthopedic Division, Federal University of São Paulo, No. 783, 04038-031 São Paulo, SP, Brazil

## Abstract

Bone marrow mesenchymal stem cells (BM-MSCs) are considered a good source for cellular therapy in cartilage repair. But, their potential to repair the extracellular matrix, in an osteoarthritic environment, is still controversial. In osteoarthritis (OA), anti-inflammatory action and extracellular matrix production are important steps for cartilage healing. This study examined the interaction of BM-MSC and OA-chondrocyte on the production of hyaluronan and inflammatory cytokines in a Transwell system. We compared cocultured BM-MSCs and OA-chondrocytes with the individually cultured controls (monocultures). There was a decrease in BM-MSCs cell count in coculture with OA-chondrocytes when compared to BM-MSCs alone. In monoculture, BM-MSCs produced higher amounts of hyaluronan than OA-chondrocytes and coculture of BM-MSCs with OA-chondrocytes increased hyaluronan production per cell. Hyaluronan synthase-1 mRNA expression was upregulated in BM-MSCs after coculture with OA-chondrocytes, whereas hyaluronidase-1 was downregulated. After coculture, lower IL-6 levels were detected in BM-MSCs compared with OA-chondrocytes. These results indicate that, in response to coculture with OA-chondrocytes, BM-MSCs change their behavior by increasing production of hyaluronan and decreasing inflammatory cytokines. Our results indicate that BM-MSCs *per se* could be a potential tool for OA regenerative therapy, exerting short-term effects on the local microenvironment even when cell:cell contact is not occurring.

## 1. Introduction

Osteoarthritis (OA) is a pathology accompanied by an increased secretion of inflammatory cytokines and proteolytic molecules into the surrounding tissue, leading to extracellular matrix degeneration and functional impairment [1]. The capacity of adult chondrocytes to maintain cartilage homeostasis declines with age, with loss of the ability to secrete the extracellular matrix components responsible for the characteristic viscoelastic properties of the cartilage [2]. Hyaluronan and aggrecan act as the major aggregating factors for collagen, proteoglycans, and water, playing a key role in the maintenance of the cartilage structure and the ability to resist to compressive loads [3, 4].

Hyaluronan is a glycosaminoglycan composed of repeated disaccharide units synthesized by hyaluronan synthases (HASs), which are membrane-bound enzymes. There are three isoforms in humans, HAS-1, HAS-2, and HAS-3, which produce hyaluronan molecules of different molecular sizes. HAS-1 and HAS-2 produce higher molecular weight hyaluronan molecules (>2 × 10^6^ Da) [5, 6]. High molecular weight hyaluronan has been described as an anti-inflammatory and immunosuppressive molecule, whereas low hyaluronan fragments exhibit immunostimulatory and proinflammatory effects [7].

Degradation of hyaluronan is regulated by hyaluronidases. There are six hyaluronidase-like sequences in the human genome [7]; however, only three hyaluronidases (HYAL-1, HYAL-2, and HYAL-3) have been described in cartilage [8]. Each enzyme acts upon molecules of different molecular weight paving the way for hyaluronan turnover in the cartilage. HYAL-1 degrades hyaluronan of all molecular weights to smaller oligomers. HYAL-2 cleaves only high and intermediate molecular weight hyaluronan yielding products of approximately 20 kDa, while little is known about HYAL-3 enzymatic activity [7, 9]. Of the three hyaluronidase genes, HYAL-2 is the most expressed in normal chondrocytes. Currently, there are indications that in OA there are lower hyaluronan levels and with altered molecular weight and that hyaluronidases are upregulated in response to inflammatory cytokines [10–12].

Inflammation has been described as an important factor in the development and progression of OA. The main proinflammatory cytokines described in the pathophysiology of OA are interleukin- (IL-) 1 beta, TNF, IL-6, and also IL-8 [13]. These cytokines contribute to the pathogenesis of OA through several mechanisms leading to a shift in chondrocytes phenotype. In an inflammatory environment chondrocytes become activated and increase further the expression of proinflammatory cytokines and factors involved in tissue catabolism, namely, matrix metalloproteinases and other proteolytic enzymes, which degrade hyaluronan, aggrecan, collagen, and fibromodulin. Such fragments of matrix components, in turn, also help maintain the production of inflammatory cytokines [1, 14]. Alternative therapies for cartilage regeneration in OA should ideally reduce inflammation and promote tissue remodeling. In this context, the use of mesenchymal stem cells (MSCs) has been pointed out as an interesting therapeutic option [14, 15], due to their distinct immunomodulatory, anti-inflammatory, and regenerative properties [16, 17]. MSCs anti-inflammatory properties might also be able to change chondrocytes phenotype, decreasing production of inflammatory molecules and favoring the renewal of extracellular components.

Indeed, interaction between MSCs and chondrocytes has been studied* in vitro*, especially in the context of cartilage development, evidencing a role of MSCs in forming cartilage tissue [18–20]. However, the effects of MSCs on OA-chondrocytes and on their capacity to repair the extracellular matrix have not yet been fully examined. Likewise, the potential effect of OA-chondrocytes on MSCs has so far been overlooked.* In vitro* coculture of MSCs and OA-chondrocytes represents a powerful approach to distinguish the contribution of each cell type and their interaction. Using cells from the same patients, we proposed to investigate the effects caused by the interaction with no physical contact between MSCs and OA-chondrocytes on the secretion of inflammatory markers and on hyaluronan synthesis.

## 2. Materials and Methods

### 2.1. Culture and Isolation of Human Bone Marrow Stem Cell and Chondrocytes

Both OA-chondrocytes and BM-MSCs were obtained from six patients undergoing total knee replacement (TKR) surgery. All patients were women (ages 63–80 years; average age: 70 years) with Grade III or IV knee OA according to the Kellgren and Lawrence classification [21]. Articular cartilage and bone marrow were harvested from the distal femur during TKR procedure. The study was carried out in full accordance with local ethical guidelines (CEP/Einstein 10/1268 Hospital Israelita Albert Einstein; CAAE: 0006.0.028.000-10) and samples were collected after obtaining written informed consent from all donors.

For isolation of chondrocytes, slices of OA knee cartilage from each donor were separately incubated in 0.25% type I collagenase in Dulbecco's modified Eagle's medium (DMEM) (Sigma, St. Louis, MO), overnight at 37°C in 5% CO_2_. The cells were then seeded onto tissue culture flasks for expansion and maintained as subconfluent monolayers in DMEM with low glucose (DMEM-LG) supplemented with 1 mM of* L*-glutamine, 10% fetal bovine serum (FBS), and 1% antibiotic-antimycotic solution (Gibco/Life Technologies, Carlsbad, CA).

A small volume of bone marrow was drawn from the distal femur to obtain BM-MSCs [22] and diluted in equal volume of phosphate buffered saline (PBS). The cells were then layered over Ficoll (density, 1.03 to 1.12 g/mL; GE) and centrifuged at 500 g for 30 minutes. Mononuclear cells were collected, seeded onto tissue flasks, and cultivated with DMEM-LG supplemented with 15% fetal bovine serum, 1 mM of* L*-glutamine, and 1% antibiotic-antimycotic solution (Gibco/Life Technologies). All incubations occurred in a 5% CO_2_ atmosphere at 37°C. Medium was replaced 3 times a week until cells reached confluence. At 80% confluence cells were harvested with a trypsin/EDTA solution (0.25% trypsin, 4 mM EDTA; Gibco/Life Technologies) and seeded onto new flasks.

BM-MSCs were expanded until the fifth passage and analyzed by flow cytometry to determine the expression profile of stem cell markers as defined by the International Society of Cell Therapy. All BM-MSCs samples expressed CD90, CD73, and CD105 on at least 95% of all cells with very low (or absent) expression of CD45, CD34, CD14, and HLA-DR. Differentiation of BM-MSCs into three cell types (adipocytes, osteocytes, and chondrocytes) was successfully achieved after culture with specific media (StemPro Adipogenesis, Chondrogenesis and Osteogenesis Differentiation Kit, Gibco/Life Technologies) and confirmation after specific staining with Oil Red for adipocytes, Alcian Blue for chondrocytes, and Alizarin Red S for osteocytes.

### 2.2. Coculture of BM-MSCs and OA-Chondrocytes

Cocultures (*n* = 6) were performed by seeding the paired BM-MSCs and OA-chondrocytes at a 1 : 1 cell ratio (50,000 cells each) from each donor. BM-MSCs were seeded onto the lower chamber of a 6-well plate and OA-chondrocytes onto Millicell hanging cell culture inserts (0.4 *μ*m pore size; Millipore, Billerica, MA, USA) in 5 mL of DMEM-LG supplemented with 10% FBS, 1 mM of* L*-glutamine, and 1% antibiotic-antimycotic solution. Controls were monocultures of BM-MSCs and OA-chondrocytes (50,000 cells each, 5 mL of medium). On days 3 and 6 both monocultures and cocultures were detached with trypsin-EDTA solution. Viable cells were counted using the Trypan blue exclusion technique using a Neubauer chamber. Total RNA from the cells harvested on both days 3 and 6 was extracted for quantitative reverse transcription-polymerase chain reaction (qRT-PCR) analysis and culture supernatants were stored at −80°C.

### 2.3. Hyaluronan Measurement

Hyaluronan measurement was performed using a highly specific enzyme-linked immunosorbent assay- (ELISA-) like fluorometric method [23]. Supernatants were boiled for 30 min in order to inactivate all proteolytic activity. Boiled sample triplicates and hyaluronan standards (ranging from 0 to 500 mg/L) were incubated in plates coated with biotinylated hyaluronan-binding protein, followed by a washing process, adding of europium-marked streptavidin, and an enhancement solution. Final fluorescence was measured in a fluorometer (Perkin-Elmer Life Sciences-Wallac Oy). Individual cell numbers were used to normalize the absolute amounts of hyaluronan of each sample, including when coculture was performed. In this last case we considered the sum of both cells types added in the coculture. Thus, data are presented as mean of hyaluronan concentration in pg/mL per cell (pg/mL/cell).

### 2.4. Inflammatory Cytokine Analysis

The concentrations of inflammatory molecules in the culture supernatants were simultaneously evaluated using the Cytokine Beads Array Kit (*Human Inflammation*, IL-8, IL-1*β*, IL-6, IL-10, TNF, and IL-12p70) (BD Biosciences) by flow cytometry (FACSAria, BD Biosciences, San Jose, CA) following the manufacturer's instructions and analyzed with FlowJo (TreeStar, Ashland, OR) and BD CBA software. Concentration was normalized by cell count in each culture group and the relative production of inflammatory cytokines was expressed as ng/mL/cell.

### 2.5. Gene Expression Analysis

Relative quantification of mRNA expression of hyaluronan enzymes, of extracellular molecules, and of inflammatory cytokines was performed using qRT-PCR. Total RNA was extracted with TriZol (Life Technologies) and a reverse transcriptase reaction (QuantiTect Reverse Transcription Kit, QIAGEN) was performed. qRT-PCR was carried out using the ABI7500 thermocycler (Applied Biosystems, Carlsbad, CA) and the Maxima SYBR Green qPCR Master Mix (Thermo Fisher Scientific Inc., Waltham, MA) for hyaluronan enzymes and extracellular molecules, according to the manufacturer's recommendations. Primer sequences are shown in Table 1. Expression of target genes was normalized by *β*-actin mRNA levels. The level of expression was then calculated as 2^−ΔΔCt^ and expressed as the mean. The results are presented as mean fold change relative to a calibration sample (Reference RNA for Real-Time qPCR, #636690, Clontech, Mountain View, CA, USA). For coculture analysis, fold change is presented as gene expression relative to each BM-MSC or chondrocyte monoculture.

### 2.6. Statistical Analysis

All data analyses were performed using GraphPad Prism version 6 (GraphPad Software, Inc., La Jolla, CA). Statistically significant differences per cell in hyaluronan (pg/mL/cell) and cytokine (ng/mL/cell) concentration were evaluated using two-way ANOVA with Tukey's post hoc test. Comparison of gene expression values between two groups was performed using unpaired* t*-test or the nonparametric Mann-Whitney test and multiple comparisons were performed with Kruskal-Wallis and Dunn's post hoc tests. Values are presented as mean ± SEM of triplicate wells. In all analyses, the level of significance was considered as *P* ≤ 0.05.

## 3. Results

### 3.1. The Number of BM-MSCs Is Decreased When Cocultured with OA-Chondrocytes

**Figure 1:**
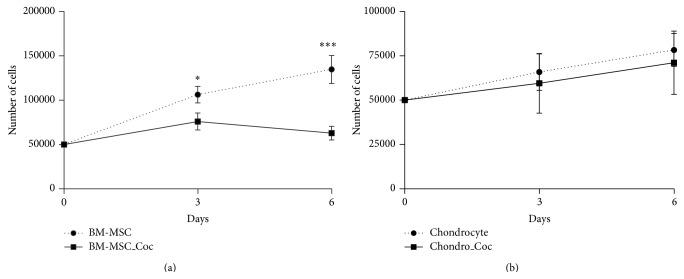
BM-MSCs decrease cell number after coculture with OA-chondrocytes. Number of cells cultivated for 3 and 6 days in monoculture (*n* = 6; bone marrow mesenchymal stem cells (BM-MSCs) and chondrocytes) or in coculture (*n* = 6; BM-MSC_Coc and Chondro_Coc). Error bars represent the SEM for the mean value. Statistical significance (two-way ANOVA) is set according to the number of asterisks, as follows: ^*∗*^ *P* ≤ 0.05, ^*∗∗∗*^ *P* ≤ 0.0001.

To determine effects of the cell coculture we counted the cell number of both BM-MSCs and OA-chondrocytes remaining at the end of the coculture and compared them to the corresponding cell number when cultured alone (Figures 1(a) and 1(b)). After three days in coculture with chondrocytes, BM-MSCs count decreased 28% compared to BM-MSCs cultured alone (7.6 × 10^4^ versus 10.6 × 10^4^). The difference increased as time in culture progressed and after six days BM-MSCs numbers were only 53% of the cells cultured alone (6.3 × 10^4^ versus 13.5 × 10^4^). We observed that the number of BM-MSCs decreased after 6 days in coculture with OA-chondrocytes in comparison to 3 days (7.6 × 10^4^ versus 6.3 × 10^4^), but no statistical difference was observed. The number of OA-chondrocytes cultured together with BM-MSCs, however, did not change significantly at both time points analyzed (Figure 1(b)). In spite of these changes, no significant variation in cell ratio was observed between BM-MSCs and OA-chondrocytes in coculture, after 3 days (75,920 BM-MSCs and 63,574 OA-chondrocytes, 1.2 : 1 cell ratio) and 6 days (65,000 BM-MSCs and 71,944 OA-chondrocytes, after 3 and 6 days, resp., i.e., 0.9 : 1 cell ratio).

### 3.2. BM-MSCs and OA-Chondrocyte Coculture Modulates Hyaluronan Production

**Figure 2:**
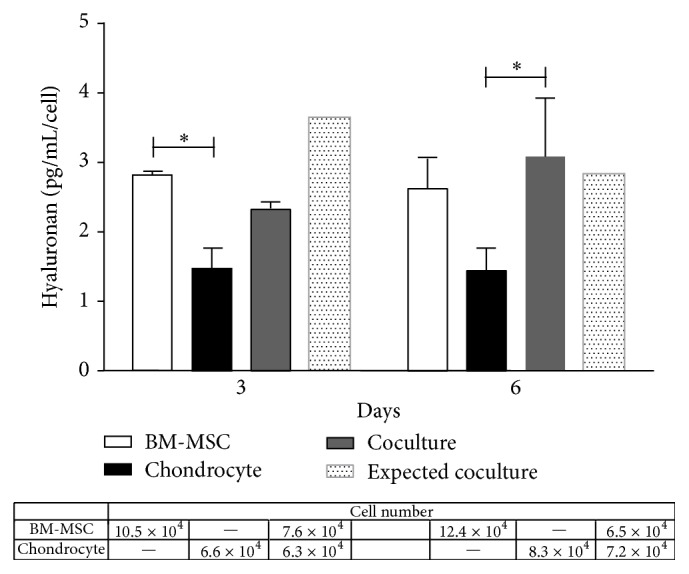
Coculture upregulates hyaluronan production. Hyaluronan concentration (pg/mL/cell) after 3 and 6 days in monoculture (BM-MSCs and chondrocytes) or in coculture. Bars represent the mean and SEM; bars with gray line (expected coculture) show “expected hyaluronan production in coculture,” based on production of monoculture and cell number counted. Table shows the cell number in each group. The asterisk (*∗*) indicates a significant (*P* ≤ 0.05) difference between cell culture groups based on a two-way ANOVA followed by Tukey's posttest.

To determine whether hyaluronan synthesis could be altered by coculture, we measured hyaluronan secreted by the cells in monoculture and when cocultured. Data on hyaluronan concentration obtained were normalized by cell number, in order to account for the greater number of cells in the coculture. To evaluate individual OA-chondrocyte and BM-MSC contribution in coculture, expected values were calculated based on the sum of values obtained from individual OA-chondrocyte and BM-MSC (monoculture) normalized by cell number of each cell type. We anticipated that the comparison between the expected and observed values could clarify whether the cross talk between OA-chondrocytes and BM-MSCs in coculture results in changes in hyaluronan production. Our results showed that both cells were able to synthesize hyaluronan, albeit BM-MSCs produced 2-fold more hyaluronan than OA-chondrocytes (2.80 pg/mL/cell versus 1.5 pg/mL/cell, resp.) after 3 days in monoculture (Figure 2).

Hyaluronan production per cell was increased in coculture when compared with OA-chondrocytes cultivated alone (Figure 2). On a per cell basis, after 6 days in our coculture system, hyaluronan present in the supernatant was 2.15-fold higher than that from OA-chondrocytes alone (3.09 pg/mL/cell versus 1.45 pg/mL/cell; Figure 2) but was similar to the levels detected in BM-MSCs monocultures (2.6 pg/mL/cell). Though, after 3 days, hyaluronan levels show intermediate values, the same is not true for values obtained after 6 days in coculture, clearly much higher. The expected values if hyaluronan production ratio was maintained after 6 days would be 2.7 pg/mL/cell (1.3 pg/mL/cell BM-MSC + 1.4 pg/mL/cell OA-chondrocyte) in contrast to the value of 3.09 pg/mL/cell observed.

Coculture is an important experiment when assessing cell:cell interactions. In our study cells were cocultured in a Transwell system that permitted harvesting of cells at the two defined time points and showed that though present proliferation rate of BM-MSCs was significantly decreased (cell number, Figure 2). On the other hand, products are continuously secreted into the supernatant making this analysis more difficult. Alternatively, in BM-MSC and OA-chondrocyte cultured alone, cell number increased, but no change in hyaluronan production was observed during the time points. In coculture hyaluronan concentration increased during time points, without changing the total cell number.

### 3.3. Hyaluronan-Related Enzymes Are Differentially Expressed in OA-Chondrocytes and BM-MSCs

**Figure 3:**
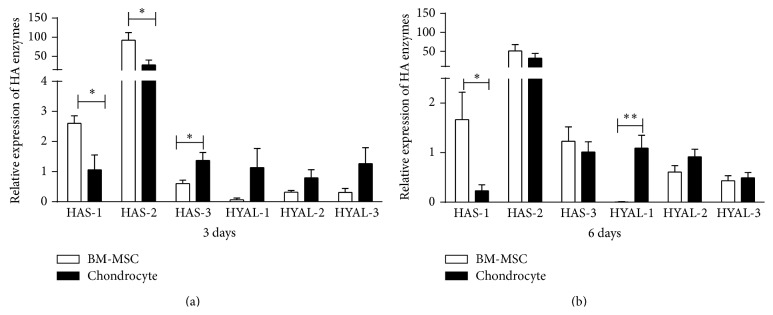
mRNA expression of hyaluronan enzymes by BM-MSCs and OA-chondrocytes. Relative mRNA expression of hyaluronan-related enzymes after 3 days (a) or 6 days (b) in monoculture. Bars represent the mean ± SEM of hyaluronan synthase- (HAS-) 1, HAS-2, and HAS-3 and hyaluronidase- (HYAL-) 2 and HYAL-3 mRNA expression. All fold changes were calculated relative to a calibrator sample. Statistical significance based on unpaired* t*-test was set according to the number of asterisks, as follows: ^*∗*^ *P* ≤ 0.05, ^*∗∗*^ *P* ≤ 0.001.

To further clarify the contribution of each cell type to hyaluronan production, we first measured hyaluronan synthase- (HAS-) 1, HAS-2, and HAS-3 and hyaluronidases- (HYAL-) 1, HYAL-2, and HYAL-3 mRNA expression in monocultures as mean fold change relative to a calibration sample. After 3 and 6 days in culture a distinct expression pattern of HAS was observed. BM-MSCs presented significantly greater HAS-1 expression than OA-chondrocytes (Figures 3(a) and 3(b)); in the latter HAS-1 was practically absent after 6 days in culture. HAS-2 also varied with higher relative values exhibited by BM-MSCs. HAS-3 gene expression was low in both cells and at both time points. On the other hand, mRNA expression of the three hyaluronidases was low at both time points (Figures 3(a) and 3(b)).

**Figure 4:**
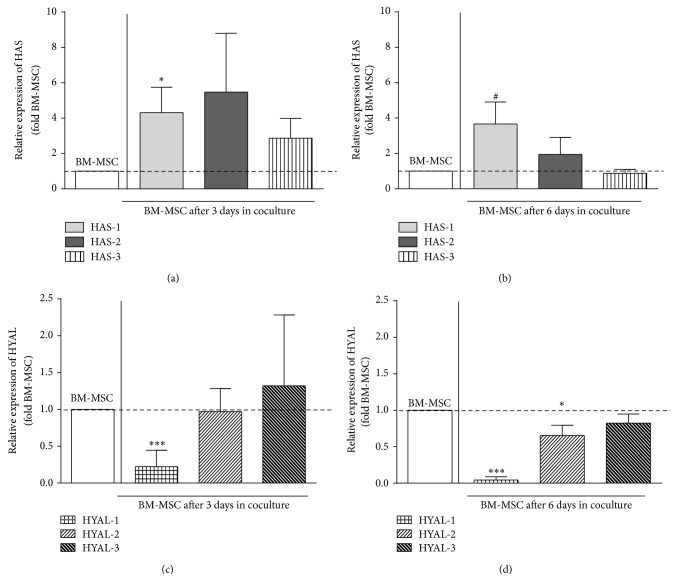
Hyaluronan enzyme mRNA expression in BM-MSC after coculture. mRNA expression of hyaluronan synthase- (HAS-) 1, HAS-2, and HAS-3 (a-b) and hyaluronidase- (HYAL-) 1, HYAL-2, and HYAL-3 (c-d) after coculture relative to time-matched and cell-matched controls. Expression of HAS in cocultivated BM-MSC relative to BM-MSC monoculture after 3 days (a) and 6 days (*n* = 5) (b); expression of hyaluronidases in cocultivated BM-MSC relative to BM-MSC monoculture after 3 days (c) and 6 days (d) (*n* = 5). Bars represent the mean fold change value ± SEM relative to BM-MSC in monoculture of hyaluronan enzymes. Statistical significance based on unpaired* t*-test was set according to the number of asterisks, as follows: ^*∗*^ *P* ≤ 0.05, ^*∗∗*^ *P* ≤ 0.001.

**Figure 5:**
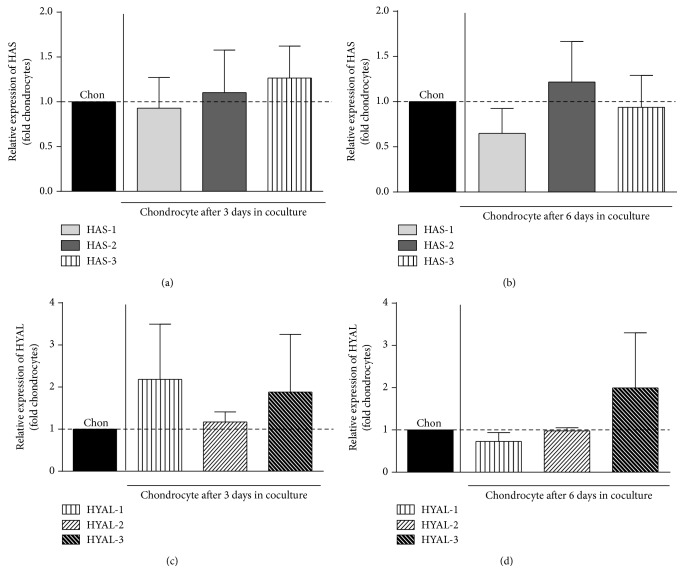
Hyaluronan enzyme mRNA expression in OA-chondrocytes after coculture. mRNA expression of hyaluronan synthase- (HAS-) 1, HAS-2, and HAS-3 (a-b) and hyaluronidase- (HYAL-) 1, HYAL-2, and HYAL-3 (c-d) after coculture relative to time-matched and cell-matched controls. Expression of HAS in cocultivated chondrocytes (Chon) relative to Chon monoculture after 3 days (a) and 6 days (b) (*n* = 5); expression of hyaluronidases in cocultivated chondrocyte relative to Chon monoculture after 3 days (c) and 6 days (d) (*n* = 5). Bars represent the mean fold change value ± SEM relative to chondrocyte in monoculture of hyaluronan enzymes. Statistical significance based on unpaired* t*-test was set according to the number of asterisks, as follows: ^*∗*^ *P* ≤ 0.05, ^*∗∗*^ *P* ≤ 0.001.

After 3 days in coculture relative HAS-1 mRNA expression by BM-MSCs was further increased compared to BM-MSCs cultured individually (3.67-fold, Figure 4(a)). After 6 days we observed a trend towards increase of HAS-1 mRNA expression by BM-MSC (Figure 4(b)). In contrast, we observed a downregulation (~25-fold) of HYAL-1 mRNA expression in BM-MSC after interaction with OA-chondrocyte at both time points (after 3 and 6 days; Figures 4(c) and 4(d)). HYAL-2 mRNA expression was also reduced after 6 days (1.5-fold) (Figure 4(d)). The expression of other enzymes was unaltered in BM-MSCs and no change in the expression of any of the enzymes was detected in the cocultured OA-chondrocytes in comparison with OA-chondrocytes in monoculture (Figures 5(a)–5(d)).

### 3.4. Gene Expression of Extracellular Matrix Components

Alteration in genes related to extracellular matrix components may reflect cartilage regeneration and differentiation status. Thus, we chose to evaluate whether coculture affects the mRNA expression of type I and type II collagen, Sox-9, and aggrecan. Our findings showed that OA-chondrocytes maintained the expression of chondrogenic markers throughout the experiment. No significant difference in expression of extracellular matrix genes (type I and II collagen, aggrecan) and Sox-9 was observed after 3 or 6 days of coculture system (Supplementary Figures  1(a) and  1(b) in Supplementary Material available online at http://dx.doi.org/10.1155/2015/640218).

### 3.5. Coculture Alters Cytokine Production

To evaluate the effects on the inflammatory microenvironment, we measured six cytokines (IL-8, IL-1*β*, IL-6, IL-10, TNF, and IL-12p70) in the culture supernatants of OA-chondrocytes and BM-MSCs cocultures. Only IL-6 and IL-8 were present in detectable levels and there was no evidence of production of the remaining cytokines investigated. Similarly to hyaluronan synthesis, we normalized cytokine production by the cell number measured at 3 and 6 days.

**Figure 6:**
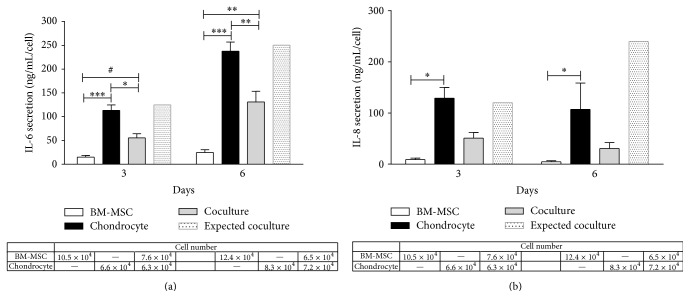
OA-chondrocytes produce higher IL-6 and IL-8 levels than BM-MSCs and cocultured cells and decrease these cytokines. Interleukin (IL) production for 3 and 6 days in monoculture (*n* = 5; BM-MSCs and chondrocytes) or in coculture (*n* = 5). IL-6 concentration (ng/mL/cell) (a). IL-8 concentration (ng/mL/cell) (b). Overall, bars represent the mean value with the SEM; bars with gray line (expected coculture) show expected ILs synthesis in coculture, based on production of monoculture and cell number counted. Table shows cell number in each group. Statistical significance based on a two-way ANOVA with Tukey's posttest was set according to the number of asterisks, as follows: ^*∗*^ *P* ≤ 0.05, ^*∗∗*^ *P* ≤ 0.001, and ^*∗∗∗*^ *P* ≤ 0.0001. Tendency of statistical differences between cell culture groups was identified by #.

As expected, OA-chondrocytes produced 8-fold greater amounts of IL-6 than BM-MSC after 3 days (113 ng/mL/cell versus 14 ng/mL/cell), a difference maintained at 9-fold after 6 days (238 ng/mL/cell versus 25 ng/mL/cell; Figure 6(a)). Similarly, OA-chondrocytes produced more IL-8 than BM-MSCs, a 14-fold increase after 3 days (130 ng/mL/cell versus 9 ng/mL/cell) and a 21-fold increase after 6 days (107 ng/mL/cell versus 5 ng/mL/cell; Figure 6(b)). Interestingly, as a result of coculture IL-6 secretion per cell was of lower levels when compared to chondrocytes in monoculture (55 ng/mL/cell versus 113 ng/mL/cell) and of greater levels when compared to BM-MSCs (55 ng/mL/cell versus 14 ng/mL/cell). This pattern was also observed after 6 days (130 ng/mL/cell versus 238 ng/mL/cell, coculture versus OA-chondrocyte, and 25 ng/mL/cell BM-MSC, resp., Figure 6(a)). In contrast to the increase in hyaluronan observed, the secretion of IL-6 by OA-chondrocytes was clearly downregulated. The expected values if secretion levels were maintained would be 249 ng/mL/cell (12 ng/mL/BM-MSC + 237 ng/mL/OA-chondrocyte = 249 ng/mL/cell) but were only 130 ng/mL/cell. The same occurred with IL-8 where expected values would be 109 ng/mL/cell (i.e., 2.3 ng/mL/BM-MSC + 107 ng/mL/OA-chondrocyte) but reached only 30 ng/mL/cell, indicating a trend for lower production.

IL-8 measured after coculture was also lower than when OA-chondrocytes were cultured individually but did not reach statistical difference after both 3 (130 ng/mL/cell versus 51 ng/mL/cell) and 6 days (107 ng/mL/cell versus 30 ng/mL/cell, Figure 6(b)). Although the IL-8 concentration in coculture observed (30 ng/mL/cell) was different from the expected (109 ng/mL/cell, i.e., 2.3 ng/mL/BM-MSC + 107 ng/mL/OA-chondrocyte), we did not reach statistical difference between coculture and OA-chondrocytes.

## 4. Discussion

In the present study we sought to establish the impact of autologous BM-MSC on hyaluronan production and their effects on the secretion profile of chondrocytes from patients with OA. Our coculture experiments suggest that an interaction occurs between BM-MSC and OA-chondrocytes in a Transwell system, which favors hyaluronan production. Moreover, we were able to show that BM-MSCs alone produce high amounts of hyaluronan and exhibit abundant HAS-1 mRNA expression.

Hyaluronan is a key component of the cartilage matrix and is used widely as an anti-inflammatory and antinociceptive agent in the treatment of OA, improving joint lubrication and shock absorbance [24, 25]. Intra-articular hyaluronan injection has been employed in the management of patients with OA. The anti-inflammatory, anabolic, and chondroprotective action of hyaluronan has been increasingly evidenced, suggesting that hyaluronan helps to reduce pain and improve cartilage function [26]. Therefore, insights into mechanisms that can change hyaluronan levels in OA are relevant.

Several studies have investigated the effects of MSCs on chondrocytes [18, 27–29], but few have provided data to show that MSCs, and not only chondrocytes, might also be affected by the cell:cell interactions. Our study shows that coculture of BM-MSCs with OA-chondrocytes led to a decrease in BM-MSCs cell numbers which can be explained by reduced cell proliferation or cell death. However, cell death was not directly measured in the present study. These low BM-MSCs numbers after 3 or 6 days in coculture suggest that OA-chondrocytes are capable of altering BM-MSCs behavior. Similar results have been shown in studies using coculture of BM-MSCs with chondrocyte pellets from different sources, during 3- to 4-week periods of culture, in which BM-MSCs numbers decreased progressively and differentiated into chondrocytes [18, 30]. Thus, it is possible that in the present short-term cultures the trend towards differentiation into chondrocytes with a slowdown of the proliferation rate was already present and that an extended culture time would eventually favor chondrogenic marker alterations. In fact, in a previous study employing coculture with direct contact between MSCs and OA-chondrocytes, the increase in chondrogenic markers was observed later, beginning only after day 7 [31]. Nevertheless, our findings show that a short culture period is necessary and sufficient to change hyaluronan production in this microenvironment.

Hyaluronan production by MSCs has been recently described [32], but we have shown that an at least twice greater hyaluronan production occurs by BM-MSCs from the same patient than by OA-chondrocytes themselves. In addition, when BM-MSCs were cultured together with OA-chondrocytes, the pattern of increased production was still maintained although at lower rates than when BM-MSCs were cultured alone. Reduction in BM-MSC cell number concomitant with no change in OA-chondrocyte number most probably affected hyaluronan production. Given that the expected and observed values of hyaluronan showed significant differences, it can be concluded that BM-MSCs contribute to hyaluronan production in our coculture system.

Thus, the present study provides evidence for a role of BM-MSCs in hyaluronan synthesis in OA. Our values for hyaluronan production were similar to the short-term (1 day) BM-MSCs monolayer cultures (range between 2.25 and 49.74 pg/mL/cell [32]) even though we cultured the cells for longer periods (6 days).

Hyaluronan enzymes HAS-1 and HAS-2 have previously been shown to be downregulated in chondrocytes collected from OA cartilage when compared to the primary chondrocytes or cartilage [33, 34]. We measured mRNA expression levels of hyaluronan synthases to clarify which and to what extent BM-MSCs and OA-chondrocytes contribute to hyaluronan levels produced. Our data show that HAS-1, but not other isoforms, is increased in BM-MSCs when compared to OA-chondrocytes, suggesting that HAS-1 may have an important role in the hyaluronan production in our system. HAS-1 is the preponderant hyaluronan synthase present in native cartilage, which synthesized the high molecular weight hyaluronan which is found in normal cartilage [33]. The greater HAS-1 expression in BM-MSCs after coculture with OA-chondrocytes indicates that BM-MSCs might be capable of upregulating hyaluronan production in OA cartilage, though. The cocultured OA-chondrocytes exhibited unchanged expression of HASs after 3 or 6 days in culture. It is possible that 6 days is not enough time to induce this change. It is also important to note that a 3D culture could eventually induce more rapid changes as a result of the BM-MSCs secreted factors.

Hyaluronan levels in the extracellular matrix and, ultimately, the regenerative potential in cartilage are determined not only by hyaluronan synthesis and but also by its degradation. Therefore, expression of enzymes involved in hyaluronan degradation was analyzed in OA-chondrocytes and in BM-MSCs. Among these enzymes, HYAL-1 has been described as an enzyme critical for cartilage development [8]. HYAL-1 mRNA was detected at higher levels in OA-chondrocytes than in BM-MSCs cultured alone. To our knowledge, the present study is the first to show mRNA expression profile of HYALs in BM-MSCs in comparison with OA-chondrocytes. Our results show that HYAL-1 and HYAL-2 mRNA expression are downregulated in BM-MSCs after coculture with OA-chondrocytes, in concordance with the higher amounts of hyaluronan found in this system. Because HYAL-1 and HYAL-2 hydrolyze hyaluronan fragments of different sizes and have been suggested as the most abundant hyaluronan-degrading enzymes, these enzymes may be working together to degrade hyaluronan in OA [10, 11]. The expression of other enzymes did not change in BM-MSCs and OA-chondrocytes after coculture. Thus, our data suggest that OA-chondrocytes modulate BM-MSCs by increasing HAS-1 and inhibiting HYAL-1 and HYAL-2 expression in order to synthesize higher molecular weight hyaluronan and, consequently, improve the local microenvironment.

The beneficial effect of hyaluronan on cartilage regeneration was demonstrated in an* in vivo* study using hyaluronan hydrogel combined with MSC [35]. The hyaluronan production by BM-MSCs might also have a direct anti-inflammatory role. Hyaluronan injection in the knees of OA patients has been associated with decreased IL-6, but not with IL-8 levels in the synovial fluid, which correlated with clinical improvement [36]. Another study suggested that the presence of hyaluronan reduces TNF-*α* and IL-6 concentration in coculture of OA-cartilage explants with synoviocytes [37].

Osteoarthritic cartilage is typically characterized by the presence of cytokines associated with inflammation, such as interleukin-1 beta (IL-1*β*), IL-10, IL-6, and TNF-*α*, besides proteolytic molecule MMPs. These cytokines are secreted by chondrocytes and contribute to OA development [13, 38]. Our results show that OA-chondrocytes in culture maintain secretion of high levels of inflammatory molecules even in the presence of interfering factors such as fetal calf serum. These OA-chondrocytes produced large amounts of IL-6 and IL-8 even though kept for long periods in culture, suggesting that they preserve an “inflammatory memory.” In contrast, BM-MSCs obtained from the same individual showed low levels of IL-6 and IL-8 production. In fact, coculture of OA-chondrocytes with the paired BM-MSCs reduced IL-6 secretion on a “per cell” basis. These observations are consistent with a report showing an anti-inflammatory effect of adipose-derived allogeneic MSC on OA-chondrocytes with a decrease of IL-6 and IL-8 production [27]. A differential production of IL-8 was, however, not detected in our analyses.

The beneficial hyaluronan production and anti-inflammatory role of BM-MSCs indicate that the cross talk with OA-chondrocytes may stimulate synthesis of other soluble molecules creating a more propitious environment for cartilage regeneration. Experimental models that permit cell contact (using OA cartilage explants) suggested that the microenvironment of OA cartilage does affect the chondrogenic potential of BM-MSCs [39].

The fact that in our study coculture was established without cell:cell contact opens new avenues of cell therapy using even allogeneic MSCs, which would induce short-term changes, by adding hyaluronan and blockading IL-6 and IL-8, to induce a more regenerative and less inflammatory microenvironment in the affected OA cartilage.

## 5. Conclusion

BM-MSCs produce hyaluronan and modulate this production in response to cross talk with OA-chondrocytes.

In conclusion, our data support the hypothesis that BM-MSCs produce hyaluronan in response to OA-chondrocytes, increasing mRNA expression of HAS-1 associated with HYAL-1 downregulation and hyaluronan synthesis. The interaction promoted also an overall lower IL-6 production. Taken together, these results indicate that BM-MSCs* per se* can be a potential tool for OA regenerative therapy. Our study offers insights into the mechanisms whereby treatment with BM-MSCs would exert beneficial effects on the diseased cartilage as a therapeutic strategy to increase hyaluronan production and decrease inflammation locally. More importantly, our data point to a strategic role of MSCs in differentiating into more active, specialized cells and not only in remodeling chondrocytes. However, more basic and preclinical studies that consider MSC as an alternative OA treatment are needed.

## Supplementary Material

Supplementary Table 1: summarizes the raw data of hyaluronan, IL-6, IL-8 and cell number measured after 3 and 6 days in culture.
Supplementary Figure 1: shows the mRNA expression of genes related to the extracellular matrix (type I and II Collagen, Aggrecan) and Sox-9, a cartilage specific transcription factor, in cells cultivated in monoculture and after coculture. During the analyzed time, we did not observe significant differences in expression of these selected genes (P>0.05).

## Figures and Tables

**Table 1:** Sequence of primers.

Gene name	Sequence
Beta-actin	5′-GGCACCCAGCACAATGAAG-3′
	5′-CCGATCCACACGGAGTACTTG-3′

HAS-1	5′-CAAGGCGCTCGGAGATTC-3′
	5′-CCAACCTTGTGTCCGAGTCA-3′

HAS-2	5′-CAGACAGGCTGAGGACGACTTTAT-3′
	5′-GGATACATAGAAACCTCTCACAATGC-3′

HAS-3	5′-GGCGATTCGGTGGACTACAT-3′
	5′-CGATGGTGCAGGCTGGAT-3′

HYAL-1	5′-GGTGAGCTGGGAAAATACAAGAA-3′
	5′-GCCCCAGTGTAGTGTCCATATACTC-3′

HYAL-2	5′-GGCGCAGCTGGTGTCATC-3′
	5′-CCGTGTCAGGTAATCTTTGAGGTA-3′

HYAL-3	5′-TGTGCAGTCCATTGGTGTGA-3′
	5′-AAGGTGTCCACCAGGTAGTCATG-3′

Collagen type I	5′-CCGCCGCTTCACCTACAGC-3′
	5′-TTTGTATTCAATCACTGTCTTGCC-3′

Collagen type II	5′-CCGAATAGCAGGTTCACGTACA-3′
	5′-CGATAACAGTCTTGCCCCACTT-3′

Aggrecan	5′-TTCAGTGGCCTACCAAGTGG-3′
	5′-AGCCTGGGTTACAGATTCCA-3′

Sox-9	5′-TGCTAGAAGATGAGGCTTCTGG-3′
	5′-GGCACTTTGTCCAGACCCA-3′
