# Supplementary material for: Modulation of Hyaluronan Synthesis by the Interaction between Mesenchymal Stem Cells and Osteoarthritic Chondrocytes
Source: Stem Cells Int. 2015 Jul 26;2015:640218. doi: 10.1155/2015/640218 (PMC4529975; doi:10.1155/2015/640218)
Supplement: Supplementary file 1 — Supplementary Table 1: summarizes the raw data of hyaluronan, IL-6, IL-8 and cell number measured after 3 and 6 days in culture. Supplementary Figure 1: shows the mRNA expression of genes related to the extracellular matrix (type I and II Collagen, Aggrecan) and Sox-9, a cartilage specific transcription factor, in cells cultivated in monoculture and after coculture. During the analyzed time, we did not observe significant differences in expression of these selected genes (P>0.05). [file 640218.f1.pdf]

Supplementary:

Supplementary Table 1. Summary of raw data (mean ± SEM) of Hyaluronan, IL-6, IL-8 produced by bone-marrow mesenchymal stem cell (BM-MSC), osteoarthritic chondrocyte (Chondrocyte) after 3 or 6 days in culture. The mean cell number per group is also presented.

	Hyaluronan (ng/ml)	IL-6 (pg/ml)	IL-8 (pg/ml)	Cell number	
				BM-MSC	Chondrocyte
**3 days**					
**BM-MSC**	415.99 (± 239.98)[a]	1403.32 (±546.69)[a,b]	805.37 (± 427.92)[a,b]	$10.5x10^4$ (± 1.3 $x10^4$)	-
**Chondrocyte**	80.25 (± 41.87)[b]	10096.31 (±4978.42)	10260.29 (± 5108.93)	-	6.6 $x10^4$(± 2.8 $x10^4$)
**Coculture**	323.84 (± 162.42)	9271.97 (± 5121.84)	7137.81 (± 4420.50)	7.6 $x10^4$ (± 2.2 $x10^4$)	6.3 $x10^4$ (± 4 $x10^4$)
**6 days**					
**BM-MSC**	519.13 (± 226.01)[a]	2820.20 (± 1275.61)[a,b]	1942.09 (± 843.09)[a,b]	12.4 $x10^4$ (± 3.4 $x10^4$)	-
**Chondrocyte**	134.30 (± 64.78)[b]	23191.56 (± 10938.09)	20555.84 (± 8998.01)	-	8.3 $x10^4$ (± 3 $x10^4$)
**Coculture**	481.34 (± 253.48)	22614.08 (± 11685.04)	14893.14 (± 8243.00)	6.5 $x10^4$ (± 1.8 $x10^4$)	7.2 $x10^4$ (± 4.4 $x10^4$)

[A] DIFFERENT LETTERS MEAN P>0.001.

Supplementary Figure 1. Chondrocyte markers mRNA expression. mRNA expression of Type II Collagen, Aggrecan , Sox-9 and Type I Collagen after 3 and 6 days in co-culture relative to time-matched and cell-matched controls. BM-MSC after co-culture (a-b) and OA-chondrocyte after co-culture (c-d), (n=4). Statistical significance based on unpaired t test was set according to the number of asterisk, as follows: * $P\leq 0.05$, ** $P\leq 0.001$.

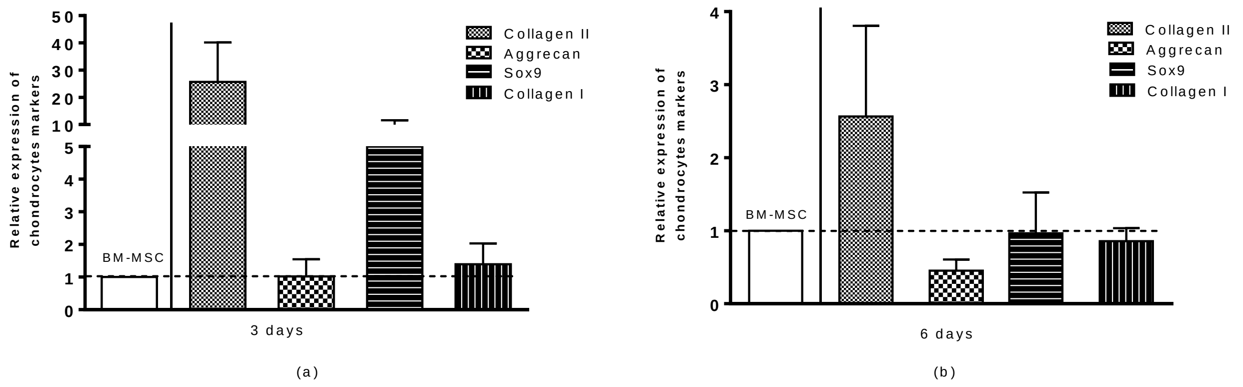

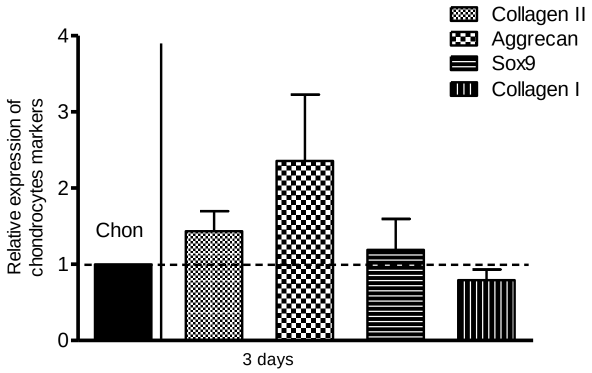
Collagen II
Aggrecan
Sox9
Collagen I
Relative expression of chondrocytes markers
Chon
4
3
2
1
0
3 days

(c)

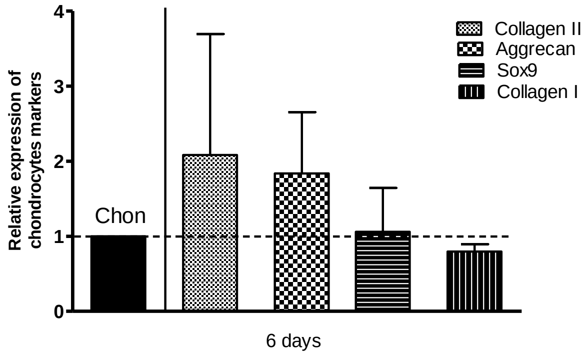
Collagen II
Aggrecan
Sox9
Collagen I
Relative expression of chondrocytes markers
Chon
4
3
2
1
0
6 days

(d)
